# Potential Role of Sulfur-Containing Antioxidant Systems in Highly Oxidative Environments

**DOI:** 10.3390/molecules191219376

**Published:** 2014-11-25

**Authors:** Emmanuel Mukwevho, Zané Ferreira, Ademola Ayeleso

**Affiliations:** 1 Department of Biochemistry, North West University, Private Bag X2046, Mmabatho 2735, South Africa; 2 Department of Biochemistry, University of Johannesburg, P.O. Box 524, Auckland Park 2006, South Africa; E-Mails: zane.ferreira@yahoo.com (Z.F.); ademola.ayeleso@gmail.com (A.A.)

**Keywords:** sulfur, antioxidant systems, oxidative environment, reactive oxygen species, glutathione, thioredoxin, glutaredoxin

## Abstract

All forms of life maintain a reducing environment (homeostasis) within their cells. Perturbations in the normal redox state can lead to an oxidative environment which has deleterious effects, especially in health. In biological systems, metabolic activities are dependent mainly on mitochondrial oxidative phosphorylation, a metabolic pathway that uses energy released by the oxidation of nutrients to produce ATP. In the process of oxidative phosphorylation, electrons are transferred from electron donors to electron acceptors such as oxygen in redox reactions and often results to the generation of reactive species. Reactive oxygen species consist of a class of radical and non-radical oxygen derivatives. The imbalance between the reactive oxygen species and antioxidant defence systems leads to oxidative burden and hence, damage biological molecules. Antioxidants help to prevent or fix the deleterious effects of reactive species. Sulfur is an important element in biological systems. This atom is usually integrated into proteins as the redox-active cysteine residue and in molecules such as glutathione, thioredoxin and glutaredoxin which are vital antioxidant molecules and are therefore essential for life. This review covers the role of sulfur containing antioxidant systems in oxidative environments.

## 1. Introduction

Oxidative phosphorylation is a metabolic process by which respiratory enzymes in the mitochondria synthesize ATP from ADP and inorganic phosphate (Pi) during the oxidation of organic molecules such as carbohydrates, fats and proteins. Oxidation is a crucial part of both aerobic life and metabolism [1] because it provides energy for the cell to perform its functions. Molecular oxygen, which is needed to sustain life* i.e.*, in human beings, can be toxic through the formation of reactive oxygen species (ROS) [2] and antioxidants are vital in curbing the toxicity which is beneficial in health and longevity. An antioxidant is simply defined as a molecule that is capable of preventing or slowing the oxidation of other molecules. It does so even at relatively small concentration, thus having diverse physiological roles in the body and health [3]. An antioxidant molecule is stable enough to give an electron to a rampaging free radical and neutralize it, hence preventing oxidative damage [4]. There is an array of naturally occurring antioxidants in nature which differ in their composition, properties (physical and chemical), mechanisms and site of actions [5,6].

The strategies of antioxidant defense mechanisms involve both enzymatic (such as superoxide dismutase, catalase, glutathione peroxidase, and glutathione reductase) and non-enzymatic (such as vitamins A, C, and E, bioflavonoids, glutathione and antioxidant minerals (copper, zinc, manganese, and selenium) [7]. Antioxidants can be classified kinetically into six categories; antioxidants that break chains by reacting with peroxyl radicals having weak O-H or N-H bonds; antioxidants that break chains by reacting with alkyl radicals; hydro peroxide decomposing antioxidants; metal deactivating antioxidants; cyclic chain termination by antioxidants: synergism of action of several antioxidants* i.e.*, phenol sulfide in which phenolic group reacts with peroxyl radical and sulfide group with hydro peroxide [8]. Furthermore, the activity of antioxidants is dependent on complex factors such as the nature of the antioxidants, conditions of oxidation, properties of the oxidizing substrate and stage of oxidation [9]. Sulfur is found in all living cells and it is a key component of some proteins which are essential for health. This review presents the potential role of sulfur containing antioxidant systems such as glutathione, thioredoxin and glutaredoxin in oxidative environments.

## 2. Oxidative Environment in a Biological System

Reactive oxygen species (ROS) is used as a collective term for reactive forms of oxygen, including both radical and non-radical species that is involved in the initiation and/or propagation of chain reaction [10]. Free radicals are molecules or molecular fragments which contain one or more unpaired electrons [11] which are very reactive and capable of engaging in rapid chain reactions that destabilize other cellular molecules resulting in production of many more free radicals [3]. Sources of free radicals include internal sources (mitochondria, xanthine oxidase, phagocytes, reactions involving iron and other transition metals, peroxisomes, arachidonate pathways, exercise, ischemia/reperfusion, inflammation), external sources (cigarette smoke, environmental pollutant, radiations, ultraviolet light, ozone, certain drugs, pesticides, anaesthetics and industrial solvents) and physiological factors (mental status and disease conditions) [10].

**Figure 1:**
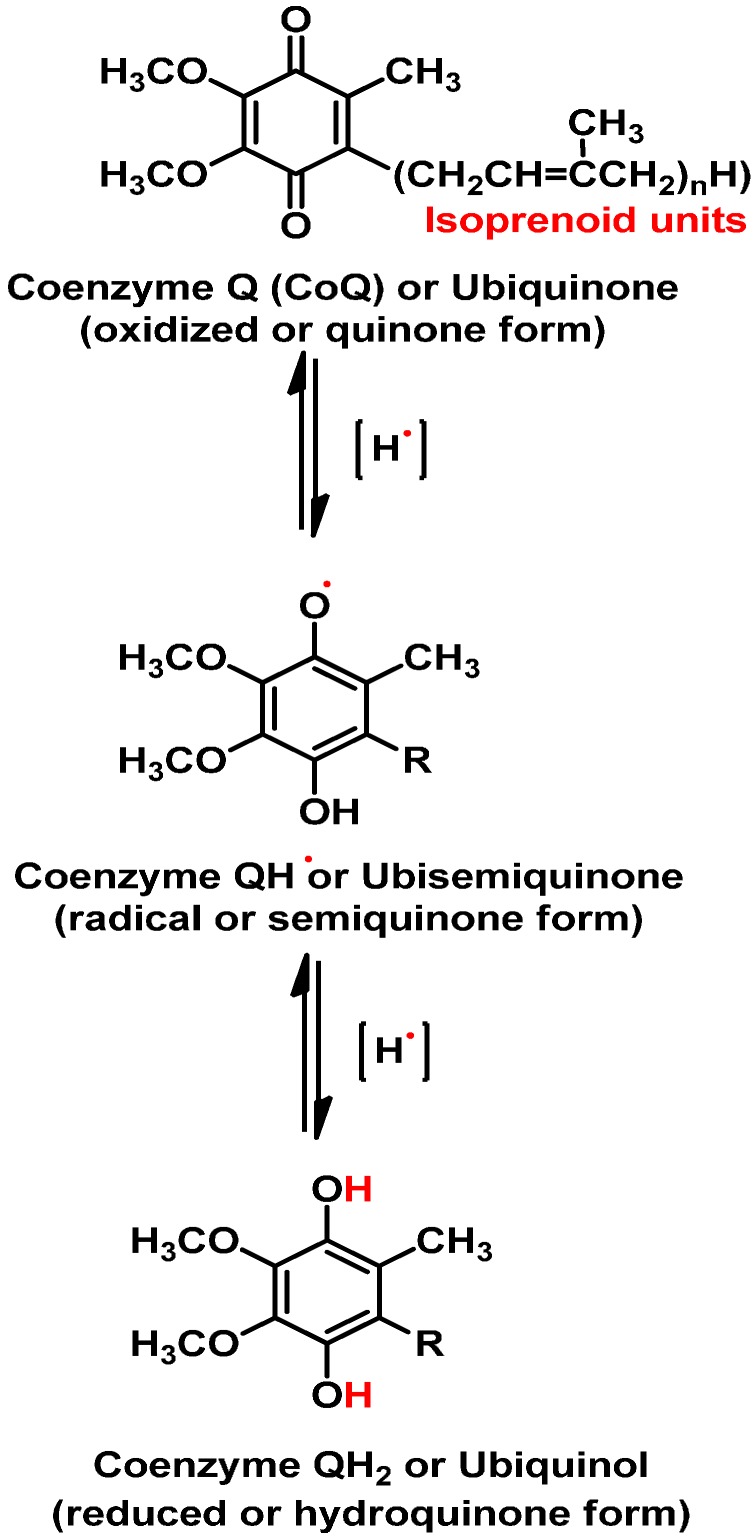
The chemical structure of Coenzyme Q (CoQ) showing the three forms it undergoes during electron transport in oxidative metabolism. The three forms are oxidized form, radical form and reduced form. CoQ (Q) + 2H^+^ + 2e^−^→CoQH2 (QH2), Eo' = + 0.060 V.

The term “oxidative stress” is used to depict an excessive amount of ROS, which is the net result of an imbalance between generation and destruction of ROS and the latter being regulated by antioxidant defences [12]. Reactive nitrogen species (RNS) is also a subset of ROS [13]. Examples of ROS and RNS include hydroxyl radical (•OH), hydrogen peroxide (H_2_O_2_), superoxide (O_2_•-), nitric oxide (NO•), peroxynitrite (ONOO-) [14,15]. Components of electron transfer that are abnormally reduced by respiratory inhibition* i.e.*, ubisemiquinone, in particular reacts directly with oxygen to form toxic radicals [16]. Ubisemiquinone is generated when one electron is unpaired during the transition of coenzyme Q (CoQ) from oxidized form to reduced form (Figure 1). For example, a mobile electron carrier coenzyme Q can appear in three forms, namely, oxidized, radical and reduced forms (Figure 1). The ubisemiquinone radical transfers a single electron to oxygen to form superoxide on the outside of the mitochondrial inner membrane [17,18,19].

Species that are less reactive such as peroxides are much more selective in the targets that they damage, with modifications occurring to specific molecules at particular sites [20]. The factors determining the extent of damage to particular targets include: (a) the target concentration; (b) the rate constant for reaction of oxidant with the target; (c) target location when compared to the site of oxidant formation; (d) secondary damaging events (chain reactions and damage transfer processes); (e) oxidant-scavenging reactions and repair reactions [20]. The induction of oxidation by ROS can lead to disintegration of cell membrane, membrane protein damage and DNA mutation [21,22]. Cellular membranes are in particular, exposed to oxidation due to their high concentrations of unsaturated fatty acid [23]. As a result of oxidative stress, the reactive intermediates produced can react with the polyunsaturated fatty acids of lipid membranes and cause lipid peroxidation [24].

Protein oxidation is caused as a result of reactions between protein amino acid residues and ROS or RNS [25]. Proteins are a major target for oxidants as a result of their abundance in biological systems, and their high reaction rate constants [20]. Oxidative cleavage of DNA can lead to the damage of all four nucleobases or the deoxyribose sugar and the mechanism of oxidative cleavage occurs in three ways which include hydrogen abstraction, addition and electron transfer [26]. Distribution of oxidative damage in the genome is dependent on the varying susceptibility of sequences to oxidative attack and the preferential targeting of repair processes [27]. ROS induced oxidative damage could further initiate and propagate the development of many diseases, such as arthritis, cancer, liver injury and cardiovascular disease [3,21,22,28].

In our previous studies, we investigated the role of antioxidant-rich natural plant products and newly synthesized novel thiazolidinediones (TZDs) hybrid compounds in relation to diabetes mellitus and obesity [28,29]. From our results, an increased lipid peroxidation due to the production of ROS in diabetic conditions was shown [28]. The results revealed a significant increase in the level of lipid peroxidation in the blood of diabetic control rats when compared with the normal control rats. Also, the blood antioxidant capacity (ORAC, oxygen radical absorbance capacity) was also significant reduced in the untreated diabetic rats. The natural products (red palm oil and rooibos) used in the study showed protective effects against oxidative stress by improving the antioxidant status. Furthermore, the hybrid compounds improved the expression of genes that are associated with lipid metabolism [29]. In our recent studies, new synthesized TZDs hybrid compounds have also shown good antioxidant activities that could be helpful in combating the deleterious effects of ROS (unpublished data).

## 3. Sulfur as An Essential Element in Biological System

**Figure 2:**
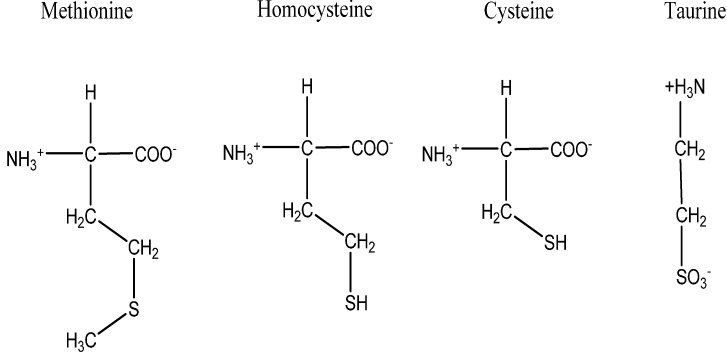
The structures of sulfur containing amino acids: methionine, homocysteine, cysteine and taurine.

Sulfur, a chemical element with symbol (S) is an abundant and multivalent non-metal. In the solid form, it is yellow, brittle, odorless, tasteless, and insoluble in water [30]. Sulfur is an important element for the entire biological kingdom due to its incorporation into proteins and other biomolecules [31]. Four common sulfur-containing amino acids are methionine, cysteine, homocysteine and taurine as shown in Figure 2 but principally among them, methionine and cysteine are among the twenty canonical amino acids that are incorporated into proteins [32]. Sulfur-containing amino acids are more abundant in animal and cereal proteins than in legume proteins, with the ratio of methionine to cysteine appearing to be higher in animal proteins than in plant sources [30]. Taurine (2-aminoethanesulfonic acid) is also an amino acid found in substantial amounts in mammalian tissues [33] and can act as an antioxidants [34].

There are three main forms of organic sulfur in animals and humans and they include; thiomethyl of methionine residues in protein; the sulfhydryl disulfides of protein and compounds containing ester or amide bound sulfates of glycosaminoglycans, steroids, and many xenobiotic metabolites [35]. Sulfur can either be incorporated as sulfate in a reaction referred to as sulfation or it is first reduced to sulfide, the substrate for cysteine synthesis [36,37,38]. In plants, the majority of the sulfur is assimilated in the reduced form [38]. Biosynthesis of organic sulfur compounds from sulfate occurs primarily in plants and bacteria while the oxidation of these compounds to sulfate takes place in animal species [30]. Sulfate is a key anionic solute and is needed for the synthesis of many different metabolites and coenzymes [38]. Unlike humans, plants can use inorganic sulfur to synthesize sulfur-containing amino acids hence, they are an essential source of sulfur for humans [31,35].

## 4. Sulfur Containing Compounds and Their Antioxidant Potentials

Sulfur is an essential part of many enzymes and antioxidant molecules like glutathione and thioredoxin. Some sulfur-containing compounds can efficiently form a line of defense against reactive oxygen and nitrogen species [39]. They are well known in the treatment of oxidative stress induced pathological disorders [33]. Disulfide bonds are usually found in proteins that are localized in the extracellular environment or within proteins that are on the cell surface. These disulfide bonds occur because of the oxidizing conditions in the extracellular environment. In the intracellular environment however, reducing conditions are maintained and disulfide bonds are absent. There are a number of proteins that are responsible for maintaining this reducing environment and these include glutathione, thioredoxin and glutaredoxin [40].

### 4.1. Glutathione Antioxidant System

**Figure 3:**
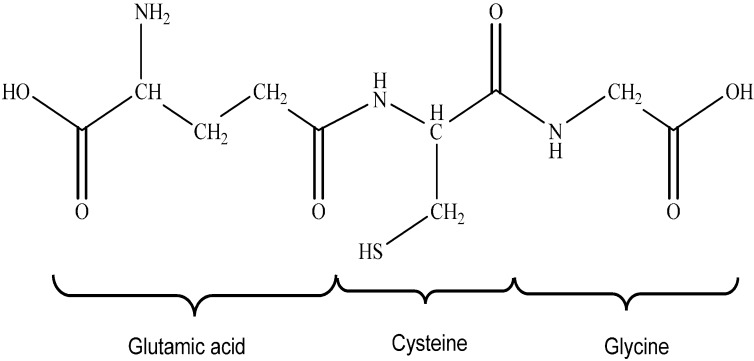
The structure of glutathione.

Glutathione (GSH) is a tripeptide, L-γ-glutamyl-L-cysteinylglycine and has a molecular weight of 307 g/mol [41]. The structure of glutathione is shown in Figure 3. The unique peptide γ-linkage protects the protein from being degraded by aminopeptidases. GSH is present in different forms including glutathione disulfide (GSSG), which is formed as a result of oxidation, and the mixed glutathione type, GSSR which are glutathione-cysteinyl disulfides [41,42]. GSH is found in the cytosol, mitochondria and endoplasmic reticulum of eukaryotic cells; however most of the GSH (80%–85%) is present in the cytosol [42]. The GSH system is one of the major thiol-dependant antioxidant systems in mammalian cells while GSH specifically is the most abundant non-protein thiol in mammalian cells [43]. Both GSH and GSSG act in concert with other redox-active compounds *i.e.*, NADPH to help in the regulation and maintenance of cellular redox status and the estimated redox potential for the GSH/GSSG couple in most cells and tissues ranges from −260 mV to −150 mV [44].

The rate of glutathione synthesis is decreased with aging and decreased GSH synthesis have been found in diseases such as diabetes, cystic fibrosis and a variety of hepatic disorders. Decreased GSH synthesis occurs as a result of decreased expression of the synthetic enzymes. In some cases polymorphisms of the different subunits of the biosynthetic enzymes can reduce the synthesis of GSH through decreased activity of the synthetic enzymes [42]. Since GSH is involved in the removal of reactive oxygen species (ROS) from the cells, a decreased production of GSH will lead to an accumulation of ROS in the cell. ROS can cause DNA, protein and membrane damage thus a decreased expression of GSH has been linked to atherosclerosis, HIV, certain cancers and rheumatoid arthritis [45]. On the contrary, an increased production of GSH has been associated with drug and radiation resistance in the treatment of certain cancers [46]. The synthesis of GSH occurs through two consecutive ATP-requiring steps. The first step is done through the action of glutamate-cysteine ligase which was formerly known as γ-glutamylcysteine synthetase, leading to the formation of γ-glutamyl-cysteine from glutamate and cysteine. The second reaction requires the action of glutathione synthetase and during this step a glycine molecule is added to the γ-glutamylcysteine to form GSH [42,45]. GSH can also be degraded into it constituent amino acids through the action of γ-glutamyltranspeptidase and cysteinyl-glycinedipeptidase [41].

#### Glutathione Synthesis

The first step of GSH synthesis that is catalysed by glutamate-cysteine ligase is the rate limiting step. This enzyme is regulated through non-allosteric feedback inhibition by GSH and also by the availability of cysteine, a rate limiting substrate during this reaction. The cysteine that is found in the extracellular environment is almost always in an oxidized state, cystine, and is the only source of intracellular cysteine that is needed for GSH synthesis. Cystine is taken up by cells and is rapidly reduced to cysteine for GSH biosynthesis. The availability of cysteine in hepatocytes is regulated by the membrane transport of cysteine, cystine and methionine [31,42].

Glutathione synthetase, which catalyses the second step of GSH biosynthesis, is not regulated through feedback inhibition. Studies have shown that overexpression of glutathione synthetase had no significant effect on the GSH levels [47]. It is generally thought that glutathione synthetase plays no role in the regulation of GSH synthesis however; it might function in the regulation of the overall synthesis of GSH in certain tissues [42]. Glutathione peroxidases use glutathione as a co-substrate and are responsible for the reduction of H_2_O_2 _to water. Glutathione peroxidases can also reduce lipid hydroperoxides and other soluble peroxides. It is found in the cytosol, mitochondria and peroxisomes of cells. The metabolism of H_2_O_2 _catalysed by glutathione peroxidase occurs through a bi-substrate enzymatic reaction. Firstly, the enzyme reacts with H_2_O_2 _and forms a selenic acid on the selenol active site of the enzyme. A molecule of GSH then reduces the selenic acid and forms a glutathiolated selenol at the active site of the enzyme. A second molecule of GSH restores the active site and results in the oxidized form of glutathione known as glutathione disulfide (GSSG) [48]. It is possible for GSSG to be reduced to form GSH again. This reaction is catalysed by glutathione reductase (GR) and requires NADPH, a reductive biosynthesis coenzyme [42].

$$\mathrm{GSSG}+\mathrm{NADPH}+H^{+}\overset{\mathrm{GR}}{\to }2\mathrm{GSH}+{\mathrm{NADP}}^{+}$$

Glutathione peroxidases are able to reduce H_2_O_2 _as well as organic peroxidases whereas glutathione S-transferases can only reduce organic peroxide. Glutathione S-transferases protects against reactive α and β-unsaturated carbonyls, epoxides and hydroperoxides which are produced in the cells during oxidative stress. These enzymes can also protect against xenobiotic compounds that are formed intracellularly due to environmental effects. During the reaction of glutathione S-transferases, a GSH molecule is conjugated to a variety of α- and β-unsaturated carbonyls and cholesterol-α-oxides. In addition to these compounds, fatty acids, cholesteryl and phospholipid hydroperoxides are reduced by glutathione S-transferases [49].

The formation of disulfide bonds are critical for protein stability and are dependent on the redox environment of the cell. The cytosol of a cell maintains a reducing environment, thus it is rare for disulfide bonds to be formed. However, when the formation of disulfide bonds does occur, it is usually non-specific and can cause irreversible damage to proteins. These disulfide bonds can be formed as a result of changes in the redox potential of the cell’s environment or through accumulation of ROS. Glutathione (Figure 3) is responsible for the reduction of these disulfide bonds that are formed in the cytoplasm. It does this through the formation of a mixed glutathione-disulfide adduct which can then be resolved again through the action of glutaredoxin [50].

### 4.2. Thioredoxin Antioxidant System

Thioredoxins (TRX) are small globular proteins with a molecular weight of approximately 12 kDa and are responsible for catalyses of disulfide/dithiol exchange in intracellular proteins [40,43]. TRXs are the major ubiquitous proteins responsible for maintaining the intracellular reducing environment [40]. The three-dimensional structure of the TRX protein was first determined by [51]. TRX contains an active site that is made up of two neighboring cysteines and takes the basic form of Cys-Gly-Pro-Cys (CGPC) [52]. It consists of five β-strands that form the core of the protein and is surrounded by four α-helices [43]. The CGPC active site that is present in all TRX proteins is present on the surface of the protein at the amino end of the α_2_-helix [53]. The midpoint potential (E_0_) for the active site of human TRX is −230 mV and TRX disulfides are at redox potentials within the range of the intracellular GSH/GSSG redox buffer [54].

Certain diseases are associated with an increase in TRX levels such as AIDS, Sjögren’s syndrome and rheumatoid arthritis. This is possibly due to the fact that TRX is an important part of the inflammatory response. The expression of TRX is increased under circumstances of oxidative stress due the antioxidant response element present in the TRX promoter. An increased expression of TRX aims to increase the amount of reduced proteins in the cytosol. When cells are experiencing oxidative stress for example through radiation, TRX translocates to the nucleus and regulates the DNA-binding of several transcription factors such as NF-κB through reduction of a vital cysteine residue [55].

**Figure 4:**
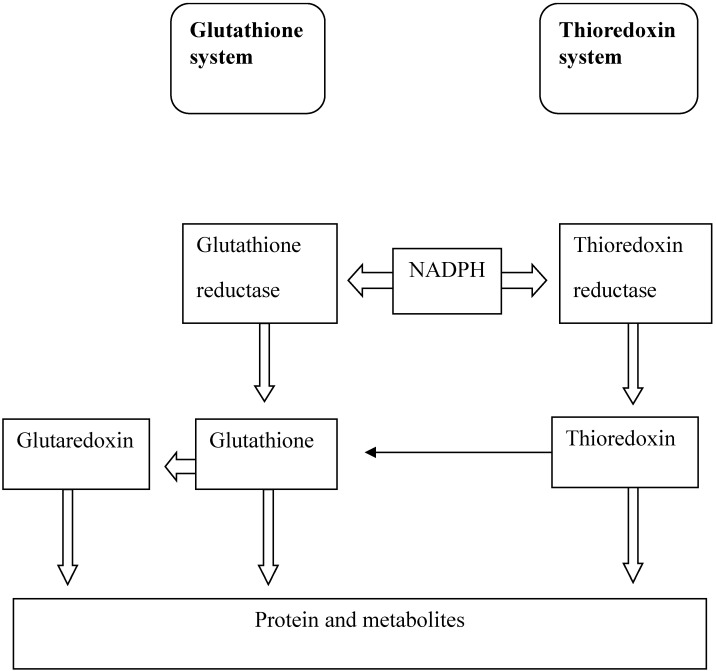
The similarities between glutathione and thioredoxin systems. The activity of both systems, is dependent on the electron donor NADPH.

TRX acts as an antioxidant through reduction of the intracellular disulfide protein bonds that are formed when ROS levels are increased. TRX can also directly decrease the levels of ROS through the enzyme thioredoxin peroxidase [55,56]. TRX catalyses the reduction of intracellular protein disulfide bonds through a bimolecular nucleophilic substitution reaction. These disulfide bonds are transferred from the protein substrate to the TRX protein through the catalytic site. The reduced form of TRX has a hydrophobic surface and binds to the target substrate protein. The thiol group of Cys32 acts as a nucleophile and attacks the disulfide bond of the substrate protein. This results in the formation of a covalent bond between TRX and the substrate protein. In the last step, the deprotonated thiol group of Cys35 attacks the disulfide of the TRX-protein complex resulting in the formation of a reduced protein and an oxidized TRX molecule [53,57]. Thioredoxin reductase is then responsible for reducing the oxidized form of TRX through an NADPH requiring reaction [52]. TRX and GSH systems have similarities in their activities as they depend on the electron donor NADPH as shown in Figure 4. TRX peroxidase is responsible for the reduction of peroxides. This enzyme is present in the cell as a dimer which is linked by two disulfide bonds. TRX reduces TRX peroxidase through the same abovementioned mechanism. The reduced form of the TRX peroxidase provides two hydrogen atoms that are then used to reduce a peroxide molecule to water [58].

### 4.3. Glutaredoxin Antioxidant System

Glutaredoxins (GRX) are glutathione dependant reductases [59]. Glutaredoxins and thioredoxins have various functions in common and also share a very similar characteristic active site of C-X-X-C. The characteristic active site of GRX is Cys-Pro-Tyr-Cys [59] compared to the TRX active site of Cys-Gly-Pro-Cys [53]. Although GRXs and TRXs share similar functions, GRXs are more flexible with regards to substrate type and reaction mechanism. Glutaredoxins form part of the TRX fold family of proteins and its three-dimensional structure consists of four internally located β-sheets surrounded by three α-helices [59]. Using a combination of redox buffers, protein-protein equilibrium and thermodynamic linkage, the human dithiol glutaredoxins (GRX1 and GRX2) with C-X-Y-C active site sequence motif have redox potentials of −232 and −221 mV respectively [60].

A variety of diseases are linked to deficiencies of specific isoforms of GRX. A deficiency in GRX1 results in mitochondrial dysfunction through the loss of mitochondrial membrane potential and this has been implicated in neurodegenerative diseases such as Parkinson’s and motor neuron diseases [61].

Loss of GRX2 function leads to decreased mitochondrial ATP synthesis caused through a dysfunction in oxidative phosphorylation [62]. GRX3 deficient cells are unable to use iron efficiently and the maturation of hemoglobin is impaired [63] while a deficiency in GRX5 prevents the formation of Fe-S clusters that are necessary for heam synthesis [64]. The expression of glutaredoxin is regulated through stress-responsive elements that are activated under circumstances of oxidative, heat and osmotic stress amongst others [65]. Glutaredoxin catalyzes the reduction of protein mixed disulfides with GSH and hence, helps to prevent oxidative stress [66]. It is responsible for the reduction of intracellular disulfide protein bonds through a reaction mechanism that is similar to that of thioredoxin. The cysteine residue that is closest to the N-terminal end of the protein nucleophilically attacks the disulfide bond of the substrate protein forming a covalent bond between GRX and the protein.

The second cysteine residue in the active site then attacks the disulfide bond of the GRX-protein complex, releasing the reduced protein molecule and leaving an oxidized form of GRX. One molecule of GSH attacks the disulfide bond of the oxidized GRX, forming a GSH-GRX complex at the N-terminal cysteine residue of the active site. A second molecule of GSH then reduces the GSH-GRX complex and yields GSSG and the reduced form of GRX. Small proteins and low-molecular weight compounds that have formed a disulfide bond with glutathione only need the N-terminal cysteine residue for reduction of the disulfide bond [59,67]. The GRX molecule has a preference to release the non-GSH as the leaving group [59] thus resulting in the mixed disulfide between GSH and GRX and a reduced form of the protein.

GRX-S_2_ + 2GSH→GRX-(SH)_2_ + GSSG

As mentioned previously, GSH can be regenerated from GSSG by the enzyme glutathione reductase.

## 5. Conclusions

Reactive oxygen species are produced as a result of oxidative respiration. If these highly reactive molecules are not kept under control through antioxidant systems, oxidative damage to DNA, proteins and membranes can occur, which has serious implications in normal cellular functions and health. Some sulfur-containing antioxidant systems as discussed in this review function to decrease the levels of harmful ROS and help to reduce intracellular protein disulfide bonds which are formed as a result of increased ROS levels. Glutathione is an example of a thiol-containing non-protein that works in concert with glutathione peroxidases and glutathione S-transferases in the reduction of peroxides. Glutathione also forms mixed glutathione-adducts which ultimately aim to reduce intracellular disulfide bonds. Thioredoxin and glutaredoxin are examples of thiol-containing proteins that are also responsible for the reduction of disulfide bonds intracellularly through the formation of a thioredoxin/glutaredoxin-protein intermediate. Thioredoxin can also directly decrease the ROS levels through the action of thioredoxin peroxidase. Therefore, sulfur containing antioxidants are essential in the maintenance of normal well-being of the cell and health.
